# Farming with Alternative Pollinators benefits pollinators, natural enemies, and yields, and offers transformative change to agriculture

**DOI:** 10.1038/s41598-021-97695-5

**Published:** 2021-09-14

**Authors:** Stefanie Christmann, Youssef Bencharki, Soukaina Anougmar, Pierre Rasmont, Moulay Chrif Smaili, Athanasios Tsivelikas, Aden Aw-Hassan

**Affiliations:** 1 International Center for Agricultural Research in Dry Areas (ICARDA), POB 6299, 10112 Rabat, Morocco; 2 L’institut Agro/Montpellier SupAgro, 2 place Pierre Viala, 34060 Montpellier, France; 3 University of Mons, Zoologie, Pentagone, Av. Champ de Mars 6, 7000 Mons, Belgium; 4 INRA, Regional Center of Agricultural Research of Kenitra, POB 257, 14000 Kenitra, Morocco; 5 Independent Consultant, 14968 138 St, Edmonton, AB T6V 1N9 Canada

**Keywords:** Ecology, Environmental social sciences

## Abstract

Low- and middle-income countries cannot afford reward-based land sparing for wildflower strips to combat pollinator decline. Two small-grant projects assessed, if an opportunity-cost saving land-sharing approach, Farming with Alternative Pollinators, can provide a method-inherent incentive to motivate farmers to protect pollinators without external rewards. The first large-scale Farming-with-Alternative-Pollinators project used seven main field crops in 233 farmer fields of four agro-ecosystems (adequate rainfall, semi-arid, mountainous and oasis) in Morocco. Here we show results: higher diversity and abundance of wild pollinators and lower pest abundance in enhanced fields than in monocultural control fields; the average net-income increase per surface is 121%. The higher income is a performance-related incentive to enhance habitats. The income increase for farmers is significant and the increase in food production is substantial. Higher productivity per surface can reduce pressure on (semi)-natural landscapes which are increasingly used for agriculture. Land-use change additionally endangers biodiversity and pollinators, whereas this new pollinator-protection approach has potential for transformative change in agriculture.

## Introduction

“Land sparing” provides direct benefit only for biodiversity, e.g. through forest areas or fallow land. “Land sharing” allows direct benefit for both, farmer and nature, e.g. through marketable flowering plants. The terms are mostly used to describe areas for nature protection on landscape level^1,2^, whereas we employ them to describe the purpose(s) of the field area necessary to sustain wild pollinators and natural enemies, two important production factors^3–9^.

State-of-the-Art pollinator protection in agricultural land are corridors spared from production and dedicated entirely to the purpose of biodiversity conservation, mainly by wildflower strips (WFS), to a lower extent by hedgerows and fallow land^4,10–13^. Farmers cannot generate income from these parts of their capital and assume the risk of spreading weeds^5^. Farmers have to invest in seeds and need a seed market for wildflowers. The European Union and USA both pursue the land-sparing approach and invest billions in agro-ecological schemes (AES) to reward e.g., farmers for seeding WFS^13,14^. The net economic impact of WFS is rarely assessed^12^. Despite the financial compensation for opportunity costs, farmers dislike WFS^12^. Pollinator decline remains high in the European Union^15–18^ and USA^19^. Pollinator protection by the European Union is described as inefficient because policies are not sufficiently targeted and harmonized^18^ and the human factor is widely overseen^20^. Low- and middle-income countries (LIC, MIC) cannot afford AES, they need economically self-sustaining approaches for pollinator protection^21^.

Farms are business entities; farmers consider benefits for future generations but increase in income is a more convincing argument^5^. The Economics of Ecosystems and Biodiversity (TEEB)^22^-approach suggests assess the economic value of ecosystem services and use it as an incentive for protection. Farming with Alternative Pollinators (FAP)^5,23,24^ is based on TEEB, assesses the value of pollination and pest control. FAP avoids high opportunity costs by using only marketable habitat enhancement plants (MHEP) and cross-sector policies to support pollinator protection in a cost-saving manner^24^. Particularly in dry regions with irrigation systems, crops can sustain natural enemies more effectively than natural habitats^25^. Wild flower visitors might benefit similarly. FAP is a targeted approach in the tradition of diversified farming systems^26^. Different to WFS, FAP includes nesting and water support out of low-cost local materials and waste products^5^. Two small pilot projects in Uzbekistan^5^ and Morocco^27^ assessed the actual impacts on net income per surface on the example of cucumber^5,27^ and sour cherry^5^ and demonstrated agronomic replicability of FAP across countries, but also the need for farmer trainings in LIC and MIC^27^.

For decades, the growing demand for pollinator dependent crops^3^ is met by intensification and expanding the cultivation areas on cost of (semi-) natural land^6,28–31^. Lack of pollinators due to agricultural intensification reduces the productivity of pollinator-dependent crops in various countries^6,9,30^. The increase of global population and a balanced diet within the boundaries of our planet^32^ can boost the demand for pollinator-dependent crops^7^ and accelerate the trend of expanding agricultural land into former (semi-) natural land^6,28^. However, many wild pollinator species depend entirely on natural land^33^. The shift to a globally scalable agricultural approach contributing to higher yields per surface and biodiversity protection is pressing, because conservation of pollinators is crucial for human wellbeing^3^. As 87% of all flowering plants require pollinators^34^ all ecosystem services highly depend on pollinators, namely those ecosystem services provided by the pollinator-dependent flowering plants^35^. Pollinator loss can cause interlinked environmental degradation and poverty spirals leading to social tension and even conflicts^35^. Therefore, both are necessary: (1) sustain diversity of crop-pollinators in agricultural land of all countries including LIC and MIC^21^ for higher productivity and food security and (2) safeguard pollinators depending on natural areas^33^ by refraining from land use changes. We assessed if FAP has potential to serve both goals and transformative change of agriculture.

Therefore, we compared FAP fields (75% of the field area is used for the main crop, 25% for MHEP and low-cost nesting and water support) with monocultural control fields concerning (1) diversity and abundance of flower visitors, natural enemies and pests, and (2) net income per surface of 100% of field areas. The trials were conducted in four considerably different Moroccan agro-ecosystems. The semi-arid region (Settat) produces mainly barley, wheat and durum and increasingly olives in monocultures. Among the four agro-ecosystems selected, wild pollinators face highest threats in semi-arid regions. The mountainous region (Sefrou in Middle Atlas) still has flowering field margins and semi-natural areas, but increasingly shifts to large orchards particularly for apple, cherry and olives and large onion monocultures. The region with adequate rainfall (Kenitra) is not far from the Atlantic Ocean, smallholder farmers produce cereals, but mostly and increasingly vegetables. Farms often have some fruit trees. The most diverse agro-ecosystem are oasis (Errachidia) with high diversity of crops in small-parceled fields. Here, alfalfa is a mass-flowering crop. In oasis, humans use the limited area with access to water for their purposes to a high extent, in consequence there are nearly no sites with wilderness or weeds as additional forage for pollinators. Protection of wild pollinators was not of concern in any of the agro-ecosystems when the project started, neither for the agricultural research and extension services nor on farmers’ level. For the assessment, if FAP has potential to reduce land-use change, the results concerning total produce and the area needed to grow this amount of food were simulated using different assumptions.

## Results

**Figure 1:**
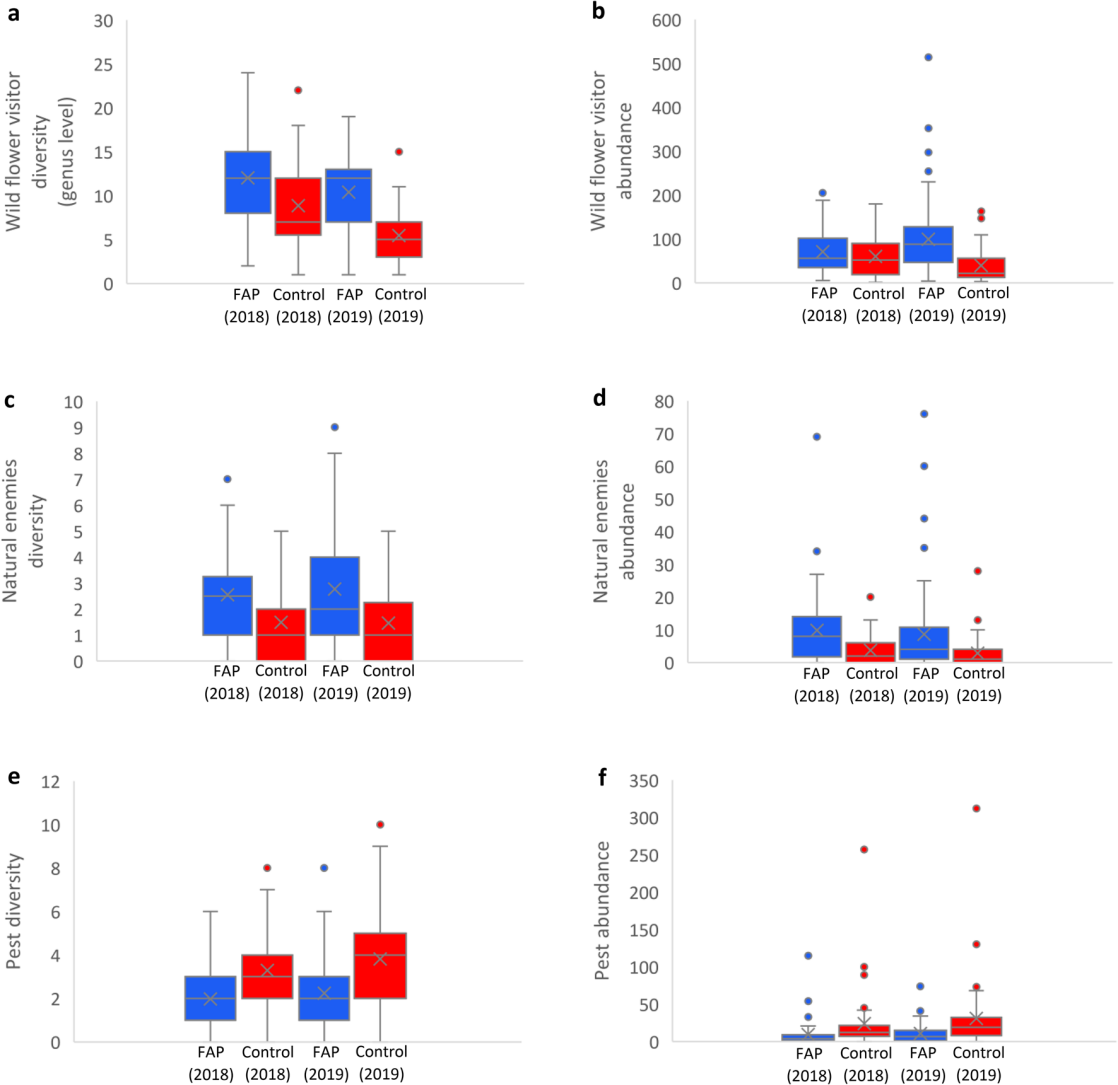
Average impact of Farming-with-Alternative-Pollinators on diversity and abundance of insects in 2018 and 2019; (**a**) diversity of wild flower visitors (genus level); (**b**) abundance of wild flower visitors; (**c**) diversity of natural enemies; (**d**) abundance of natural enemies; (**e**) pest diversity; (**f**) pest abundance.

**Figure 2:**
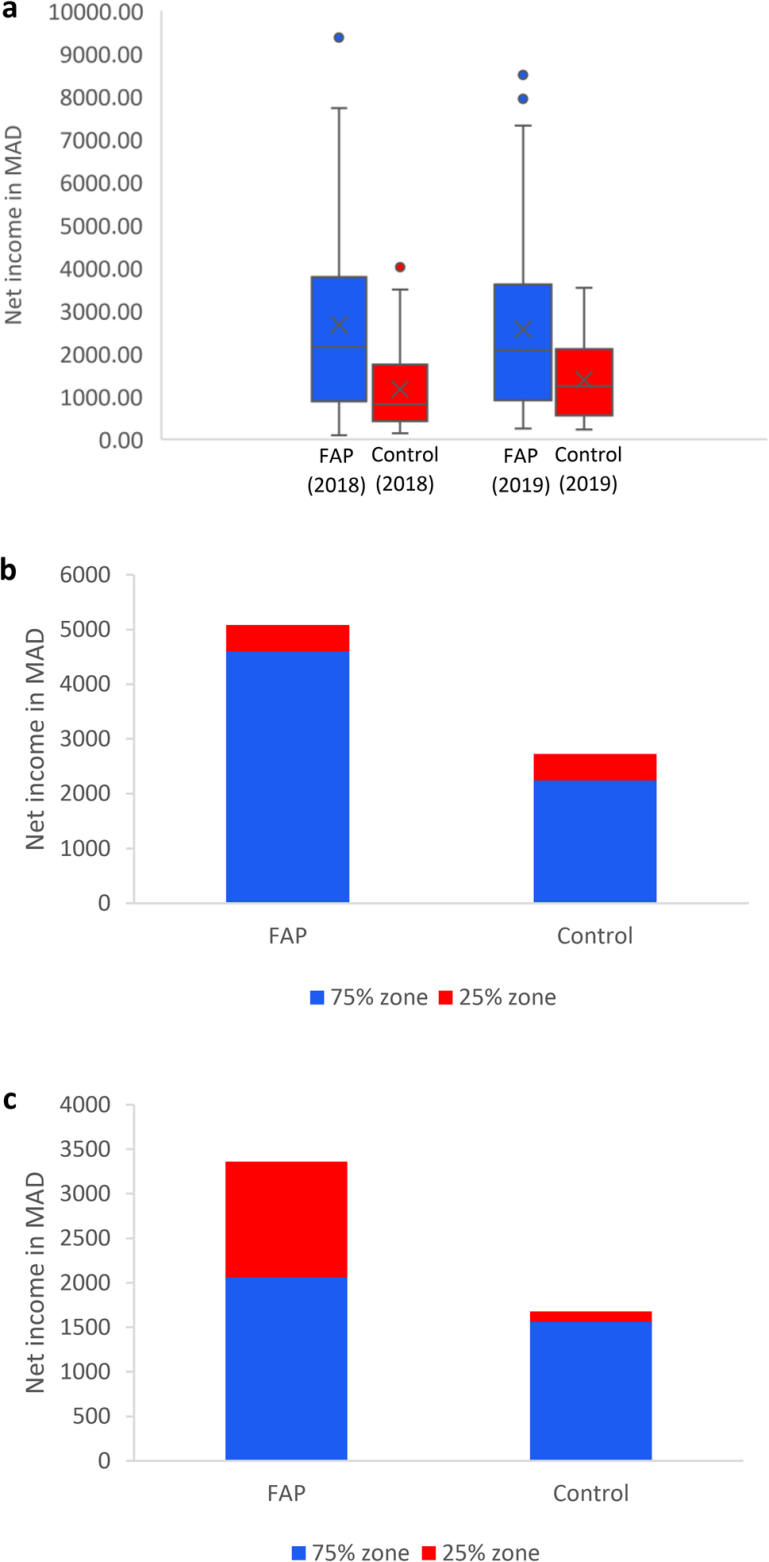
Impact of Farming-with-Alternative-Pollinators on net income per surface in Moroccan Dirham (MAD); (**a**) average net income in 2018 and 2019; (**b**) in most trials, the net-income increase results mainly from the 75% zone as in this tomato trial (2019, region with adequate rainfall); (**c**) marketable habitat enhancement plants buffer against income loss in case of threats to the main crop as in this pumpkin trial (2018, mountainous region).

For both years, our results show similar trends concerning insect diversity and abundance (Fig. 1a–f). Even on genus level we observed an overall higher taxa richness of wild flower visitors in FAP fields (W = 2836, p-value < 0.001) (Fig. 1a), and also higher abundance (Fig. 1b) compared to control fields. This includes e.g., *Amegilla* spp.*, Andrena* spp.*, Anthophora* spp.*, Bombus* spp.*, Ceratina* spp., *Colletes* spp., *Dasypoda* spp., *Eucera* spp., *Halictus* spp., *Hoplitis* spp., *Hylaeus* spp., *Lasioglossum* spp., *Melecta* spp.*, Melitta* spp.*, Osmia* spp.*, Panurgus* spp.*, Thyreus* spp.*, Xylocopa* spp., Syrphidae family, Lepidoptera order, and other minor flower visitors. The slightly mosaic planting (four to eight MHEP; supplementary data file) in the 25% zone of FAP fields also provided nectar and pollen for wild flower visitors over a longer period (on average 93 days in FAP fields; 63 days in control fields) according to flowering data in field books. Whereas control fields were attractive for beneficial insects only during the flowering of the main crop, flower visitors and natural enemies used FAP fields as forage sites also before and after the flowering of the main crop, as the insect samplings before and after the flowering time of the main crop showed. This is important for pollinator protection and pest control. The diversity (Fig. 1c) and abundance (Fig. 1d) of natural enemies was on average higher in FAP fields than in control fields, whereas pest diversity (Fig. 1e) and abundance (Fig. 1f) was on average higher in control than in FAP fields. In FAP fields, pest abundance was on average reduced by 64.8% (p-value < 0.001). The average net additional income per surface of fields was 121% (2018: 161%; 2019: 82%, Fig. 2a) in FAP compared to control fields. Average rainfall in Morocco was lower in 2019 (124.5 mm; https://fr.hespress.com/123120-pluviometrie-la-campagne-agricole-2019-2020-pas-si-verte-que-ca.html) than in 2018 (199.5 mm; https://fr.hespress.com/123120-pluviometrie-la-campagne-agricole-2019-2020-pas-si-verte-que-ca.html), which might have affected diversity and abundance of flower visitors, in particular of aquatic syrphids—and in consequence economic results. Environmental and climatic variabilities are typical under dry Mediterranean conditions. However, higher net income of FAP was significant across all locations.

The total average net income increase FAP versus control for pumpkin was 152%, for melon 61%, zucchini 111%, okra 79%, faba bean 112%, eggplant 214% and tomato 83%. The income increase was significant in all cases (p ≤ 0.05 for okra, p ≤ 0.01 for zucchini, tomato, melon and p ≤ 0.001 for eggplant, faba bean, pumpkin). Results per trial crop in the same agro-ecosystem vary partly substantially in both years concerning insect diversity and abundance. There are seasonal weather variations and natural fluctuations of insect populations, but these differences do not alter the general trend of FAP-induced higher income and the reliability of the FAP-induced incentive. The impact of pollinators on zucchini, pumpkin and melon is “essential”, on faba bean, okra and eggplant “modest” and on tomato “little”^36^, but the FAP impact on eggplant (four trials) was highest due to higher number of fruits, higher weight and better shape of fruits from FAP fields. It would require more trials to analyse, why FAP performed less with melon than with zucchini and pumpkin or why it performs extraordinary with eggplant.

In the semi-arid region FAP rose income per surface by 185%, in the mountainous region by 130%, in oasis by 88% and in the region with adequate rainfall by 80%, which shows that the FAP impact is higher in less diverse agro-ecosystems, but the incentive is reliable in the four agro-ecosystems. In 29 out of 31 trials the income from the 75% zone of FAP fields was higher than from 100% of the control fields. In 25 out of 31 trials the average income from the 25% zone in FAP was higher than from the 25% in control fields, though, based on farmers’ rankings, we used the best performing cultivars for the 25% zones of control fields. For melon, zucchini, eggplant and pumpkin the investment costs for MHEP were on average 42.96% higher than the investment in the 25% zone of control fields, but for faba bean, okra and tomato (these seeds are expensive in Morocco), the investment in seeds for MHEP was on average 39.9% lower than for control fields. Four aspects induce high income increase per surface already in the first year: the absence of relevant direct costs for seeding and the lack of opportunity costs (farmers can use the entire field for production and do not spare a part of their field for biodiversity protection as in the WFS-approach), higher diversity and abundance of wild flower visitors and lower pest abundance (Fig. 1).

The higher net income per surface is mostly based on the higher income from the 75% zones as e.g., in the tomato trial in the region with adequate rainfall 2019 (Fig. 2b). However, in the pumpkin trial in the mountainous region 2018 for example, the main crop was heavily affected by strong rainfall. In this trial MHEP contributed 38.73% (Fig. 2c) of the total additional net income, so MHEP buffered against income loss.

## Discussion

Current land-sparing protection schemes, in particular reward-based wildflower strips have been criticized as inefficient^12,18,20^ and not scalable globally^5,21^, while the urgency of pollinator protection is recognized^3,6,15–18,37^. Recent suggestions for pollinator protection focus on land-sparing with landscape approach, e.g. interconnected and well-managed habitats^38^ or mixed agricultural and silvo-pastoral landscapes^39^. These suggestions address the need to protect pollinators but not the need to increase simultaneously agricultural productivity per surface for the growing demand. We propose to discuss our results in a broader context, in particular concerning four aspects: (1) farmers’ preferences concerning plants in their fields^5,12,40,41^ and thus the probability of FAP adoption, (2) agricultural productivity^6,7,9,30,31^ as an important parameter as global population increases, (3) food insecurity^32,42,43^ and its socio-economic impacts on regional, national and global stability^35^, and (4) the potential of the pollinator-protection approach FAP to promote transformative change towards highly productive and biodiversity-oriented agriculture.

The prolonged flowering time in the fields and the diversity of flowers are important for diversity and conservation of wild flower visitors in agricultural land. Besides higher diversity and abundance of floral resources, nesting opportunities, reduced pesticides and landscape have been identified as additional crucial aspects for pollinator protection^44,45^. FAP includes—different to WFS—also nesting support out of local or waste materials e.g., for cavity nesting pollinators^5,27^. Pest abundance was on average reduced by 64.8% (Fig. 1f), so farmers have reason to reconsider if they need to invest additionally in chemicals or not. The high reduction of pest abundance is an incentive to seed MHEP and reduce chemical threats to pollinators.

The buffering effect against income loss in case a pest, disease or weather incident affect the main crop was reported also from both pilot studys^5,27^ and can become more important and valuable for farmers in the course of climate change related uncertainties. The fact that the income from the 75% zone in FAP fields was higher than the income of 100% of control fields in 29 out of 31 trials can be a convincing argument for farmers. In the FAP-sour-cherry trial in Uzbekistan the participating large-scale farmers focused on the higher productivity of the main crop and regarded the yield of MHEP as a (negligible) by-product^5^. The FAP-induced net income gain is much higher than in State-of-the-Art studies^8,10–12,46,47^ and achieved already in the first year, whereas, using WFS, it can take several years to gain net profit^46^.^.^ Not only in LIC and MIC, where AES are hardly affordable, but also in high-income countries (HIC) these two aspects, higher income already in the first year and reduced pest abundance, can motivate farmers to use FAP instead of WFS and to change their behaviour. Recent research^20^ highlights the need for incentives and ”behavioural drivers “^20^for transformative change. The absence of opportunity costs (through land sharing instead of land sparing) and the non-necessity of a seed market for wild flowering plants can additionally support change of behavior and adoption. Farmers are the decision makers on their land, pollinator protection might become more successful if farmers’ preferences for habitat enhancement plants would gain more attention^5,12,40,41^. MHEP play a most crucial role in FAP planting schemes and their environmental and economic impacts. Therefore, the impacts of different MHEP and farmers’ criteria to prioritize specific MHEP have been assessed in detail, the publication is under preparation.

Do our results have value for the broader, but often separated research areas and political discussions on food security/agronomy/agricultural research^32^ and pollinator protection/biology^15–17,37^? We want to stimulate more integrated research including both, food security/agronomy/agricultural research and pollinator ecology/pollinator protection^7,35,43,46–48^. More holistic research could promote transformative change. Therefore, we simulated potential impacts of FAP-habitat-enhancement measures concerning (1) food production/security and (2) reduced need for land-use change from semi-natural areas to cultivated areas.

**Figure 3:**
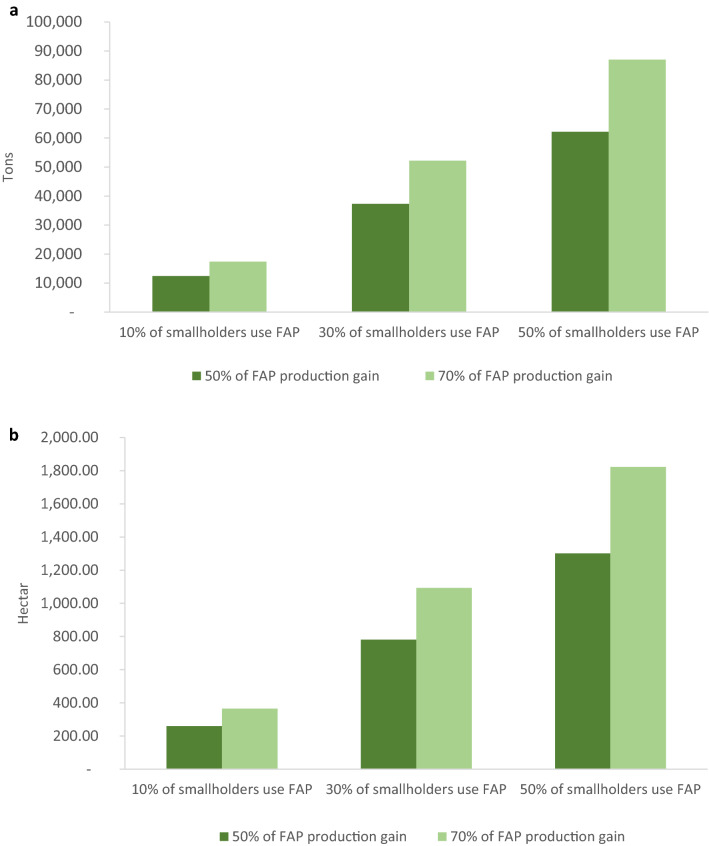
Simulation of potential impacts of Farming-Alternative Pollinators on food security and saving land for nature through smallholders based on 6 vegetables and Moroccan production data 2016–2017; (**a**) simulated potential increase of production; (**b**) simulated potential for land saving.

Worldwide, smallholder farms with less than two ha contribute 28–31% of total crop production for human consumption^49^, farms up to 50 ha provide 51–77% of all commodities and nutrients^50^. The simulations of potential FAP impacts use three different adoption rates (µ: 10%, 30% and 50%) and two different assumptions for technology effectiveness (50% and 70%). The simulations show both, (1) substantially higher food production per surface and thus higher food security (Fig. 3a) and (2) highly reduced need for agricultural land (Fig. 3b).

Even these conservative simulations of impacts of broader FAP use by Moroccan smallholders encourage introduction of FAP in more countries. Reduced land-use change to expand agricultural land would contribute to the protection of pollinators entirely depending on natural areas. The results confirm that FAP has potential to contribute to SDGs 1, 2, 3, 13 and 15^7^, that an integrated approach has advantages to meet the challenges in both areas, food insecurity and pollinator decline. The simulations unveil much higher potential for synergies between pollinator protection and food production than suggested as an outcome of WFS^4,8,46^.

FAP was developed to serve pollinator protection, food security and climate change resilience^23^. Particularly, the target higher national food security can be more attractive for strong national protagonists such as Ministry for Agriculture and agricultural extension services than a pure environmental target like pollinator protection. Thus, FAP has higher potential to promote transformative change in agricultural land (Aichi target 7) than approaches focusing on biological targets only. In Morocco, the FAP-team collaborates intensively with the national agricultural extension service (l'Office National du Conseil Agricole, ONCA) and started FAP-trials also with large-scale producers. Morocco joined the Coalition of the willing on pollinators (https://promotepollinators.org/) 10 May 2019 as first Arab country and works on a cross-sector action plan for pollinator protection.

There might be one more advantage of FAP: The rewards paid for land sparing and the seeding service (WFS-approach) do not benefit nature if farmers overuse chemicals after plant establishment. Whereas land sharing opens a dialogue with farmers based on environmentalists’ respect for farmers’ priorities^5,27^. The TEEB^22^-based FAP-approach primarily targets the human factor and a change of behavior^5,20,48^, a focus described as most important^5,20,21^. The incentive is not service related but coupled with productivity and performance: FAP rewards farmers for months of environmentally friendly performance^5,27^. This is important for pollinator conservation and transformative change. Eager to gain higher income per surface, farmers can gain motivation to observe and guard insects in their fields attentively. This might require information campaigns for farmers as additional enabling factor, as farmers’ knowledge on pollinators and pollination is limited, notably in many LIC and MIC^27^. A survey focused on knowledge necessary for FAP, which was conducted in three culturally different LIC and MIC (Benin, Turkey and Morocco) clearly showed the need for capacity building among farmers to recognize wild pollinators, nests, pollination problems etc^27^. In all countries including HIC, it is necessary to build capacity to vision multiple benefits of pollinator protection e.g., their contributions to various Sustainable Development Goals^7^ and to all ecosystem services^35^ and to realize interlinked impacts of pollinator decline and pollinator loss on ecosystems, human wellbeing, economy and conflicts^35^.

By now, pollinator decline is tackled as an environmental problem through WFS, hedgerows and fallow land, however the notion “environmental problem” reflects a “fundamental misconception” as most disturbances of ecosystems stem from maladaptive human behaviour^51^. In the Anthropocene, people have the strongest impact on our planet, therefore the Anthropocene requires pollinator protection through skilled and intrinsically motivated humans: pollinator protection not as paid environmental add-on, but as an outcome of skilled ecological society^21^. The clue to this change of paradigm and to transformative change in agriculture might be the shift from land-sparing to respectful land-sharing.

## Materials and methods

### The participants of the on-farm trials

The farmers taking part in the trials own between 0.3 and 40 ha. Most of them were smallholders (less than 2 ha) and used to plant vegetable fields of around 300 m^2^ per crop. Two out of 233 participating farmers are female, farmers’ age ranges from 24 to 68 years. All farmers learned agriculture from their parents, 70% are literate. Farmers and fields were visited 10–12 times per trial. In 2018, we started with 112 farmer fields, but some farmers did not follow strictly the obligatory agricultural practices (e.g., concerning fertilizer, irrigation, harvest), some lost the entire or parts of fields (e.g., by flood, grazing livestock), therefore all assessments concerning 2018 include 99 farmer fields. In 2019, we started with 136 farmer fields, two farmers did not follow the agreed farming practices, so assessments for 2019 are based on 134 farmer fields.

### The design of participatory field trials

We conducted 14 trials in 2018 and 17 in 2019, each trial encompasses five FAP fields and three control fields in neighbouring villages. Minimum distance between FAP fields and between FAP and control fields was two thousand metres for nearly all fields, at least more than one thousand metres. In the mountainous region we used pumpkin, zucchini and faba bean as main crops (two years), in oasis okra and zucchini (two years), faba bean and pumpkin (2019), in the semi-arid region melon, zucchini, pumpkin, eggplant and faba bean (two years) and in the region with adequate rainfall tomato, faba bean, zucchini and eggplant (two years) and pumpkin (2019). The main crops were selected by farmers and agricultural advisors of the respective regions, MHEP by farmers of the respective trials and researchers.

Field size was 300 m^2^ as recommended for smallholders^5^ with a 75% zone for the main crop in both, FAP and control. Except for okra, the 75% zone had four cultivars with four replications in a randomized system as recommended as enhanced practice by farmers in the pilot project in Morocco^27^. For okra only two cultivars are available in Morocco and trials used only seeds accessible also for farmers. FAP fields employed the 25% zones for habitat enhancement, whereas control fields had the main crop also in this zone. We used coriander, basil, cumin, dill, anise, celery, sunflower, canola, flax, zucchini, okra, melon, tomato, green pepper, cucumber, Armenian cucumber, eggplant, chia, arugula, watermelon, pumpkin, grass pea, cultivated lupinus, alfalfa, clover, vetch, faba bean and wild lupinus as MHEP, per trial between four and eight different MHEP. As faba bean starts flowering in end of February in Morocco, MHEP were partly forage crops as they flower early. MHEP were seeded in a way that around 2/3 flowered at the same time as the main crop and 1/3 before or after to prolong the foraging season on site for flower visitors. The habitat enhancement zones included also nesting and water support out of local materials, e.g., hollow stems, wood and dry mud with holes.

### Field management

In oasis, all fields were irrigated by gravity flow, in the other sites all farmers used drip irrigation. The amount of dung used is based on farmers’ decision and varies per region: semi-arid region 500 kg/300 m^2^, mountainous region 1000 kg/300 m^2^, oasis 1500 kg/300 m^2^ and region with adequate rainfall 3000 kg/300 m^2^. Soil analysis was conducted for all fields but does not explain the income gaps between FAP and control. Pesticides (mainly neonicotinoids and broad-spectrum insecticides) were prohibited during trials. In some urgent cases with permission of the plant protection specialist, one foliar insecticide application for pest management was accepted when pest density reached the economic threshold.

### Insect sampling and methods to analyse the data

The taxa richness of flower visitors was assessed by a combination of transect net samplings and pan trappings. In each field, insects were sampled four times, once before the flowering of the main crop, twice during its flowering and once afterwards. Each sampling took two days for each trial (four fields per day). Two sets of three pan traps (blue, yellow and white) were located in each field at the beginning of the first day of sampling and were collected the second day after 24 h. The samplings in 75% zones consisted of walking along two twenty eight metres transect lines for five min each. In the 25% zones flower visitors were collected once along an 80 m transect line around the 75% zone for ten minutes. The flower visitors were collected and kept separately per MHEP, but the respective time needed was recorded and added to the transect. The insects were collected using both sweep nets and insect vacuums. All flower visitors were collected except *Apis mellifera*, *Bombus terrestris* and *Xylocopa pubescens* that were identified visually on site. The collected insects were first fainted with ethyl acetate and afterwards placed inside killing jars filled with cyanide, afterwards pinned and labelled. Wild bees were identified to the genus level using the most recent key for wild bees in Europe^52^. The other flower visitors were identified to genus level or to family level. Significance concerning diversity was measured by Wilcoxon test^53^.

In the 75% zones, pest insects, predators and parasitoid wasps were collected four times. Per farmer field, four one-square-metre quadrates were randomly selected, within the quadrates ten randomly selected plants were beaten five times, so in total we used 320 crop samples per trial. In the 25% zones, the beating method was similarly used for each MHEP (five sample plants per MHEP). Specimen were collected in plastic bags and kept in plastic tubes containing 70% ethanol for conservation. Abundance of pests was estimated by counting the number (*i*) recorded on each sample crop. Pest reduction was calculated by the rate of pest reduction (%) using the following formula: % = (1− A_FAP(i)_ / A_Control(i)_) × 100, where A_FAP (i)_ is the average of the abundance in the FAP plot; A_Control (i)_ is the average of the abundance in the control plot^54^.

### Economic assessments

The economic assessments use the same calculation as the pilot projects^5,27^: the number of fruits was counted and weighed. Investment costs in FAP and control fields are the same in the 75% zones. The income from the 75% zones was assessed by multiplying total weight with market price per kg. The income from the 25% zones of control fields was assessed by total produce weight multiplied by market price per kg; investment costs were deducted. The income of the 25% zone of FAP fields was computed by multiplying total weight with market price per kg of MHEP minus respective investment costs and minus 100 MAD (1.5 person days per FAP field) as calculated labour costs for harvesting MHEP, though in our trials, farmers harvested themselves.

### Simulations

The simulation of potential FAP impacts on food security and sparing natural land for pollinator and biodiversity protection is based on following assumptions. Basis is the total production (2016–2017 differentiated per crop; provided by the Moroccan Ministry of Agriculture on request) for faba bean (share of harvested crop with green pods as in the experiments, 105,760 ton in 10,205 ha), zucchini and pumpkin (179,519 ton in 7539 ha), melon (618,588 ton in 20,163 ha), eggplant (52,966 ton in 1885 ha) and tomato (1,293,761 ton in 15,888 ha). We did not include okra due to lack of national production data. For the simulation on potential increase of production through smallholders (≤ 2 ha), we use 13% as share of smallholders in North Africa for vegetable production^49^. For the simulation of the land-saving potential through smallholders, we used 11% (North Africa, share of smallholders’ land for food crops)^55^.

The formula used for the simulation on the potential FAP impacts on food security (PIFS) is:

$${\text{PIFS}}\, = \,\left( {{\text{SSP}}*\left( {{{1}} - \upmu } \right)} \right)\, + \,\left( {{\text{SSP }}*\upmu } \right){\text{ }}*\left( {{\text{1}}\, + \,\left( {{\text{GFT }}*{\text{TE}}} \right)} \right) - {\text{SSP}}$$

PIFS: Potential increase in crop production because of FAP (t), SSP: Smallholders’ share of production in (t), GFT: FAP production gain in farm trials (%), µ: the share of smallholder-producers adopting FAP, TE: Technology effectiveness.

The GFT employed is 85,2% which represents the average FAP production gain of the vegetables used in the simulation process. For µ we used either 10%, 30% or 50% and for TE we assumed that smallholder-producers gain either 50% or 70% of the total production gain achieved in on-farm trials with smallholder-farmers since farmers will adapt MHEP and their planting to their personal preferences.

The formula used for the simulation of potential land saving (PLS):

$${\text{PLS}} = (({\text{SAP}} * {\text{PIFS}})/{\text{SSP}})-{\text{SAP}}$$

PLS: Potential land saving in ha, SAP: Smallholders’ area of production in ha.
